# DeepGaitLab: Accurate and Flexible Markerless Motion Tracking Powered by Synthetic Data

**DOI:** 10.21203/rs.3.rs-8998239/v1

**Published:** 2026-07-24

**Authors:** Soyong Shin, Sijia Li, Zhixiong Li, Anastasios Yiannakidis, Hanz Cuevas Velasquez, Chaeeun Lee, Kunwoo Lee, Julian Ng-Thow-Hing, Gelsy Torres-Oviedo, Andrea Rosso, Michael J. Black, Eni Halilaj

**Affiliations:** 1 Department of Mechanical Engineering, Carnegie Mellon University, Pittsburgh, PA; 2 Department of Electrical and Computer Engineering, Carnegie Mellon University, Pittsburgh, PA; 3 Robotics Institute, Carnegie Mellon University, Pittsburgh, PA; 4 Department of Biomedical Engineering, Carnegie Mellon University, Pittsburgh, PA; 5 Computer Science Department, Carnegie Mellon University, Pittsburgh, PA; 6 Max Planck Institute for Intelligent Systems, Tübingen, Germany; 7 Department of Bioengineering, University of Pittsburgh, Pittsburgh, PA; 8 Department of Epidemiology, University of Pittsburgh, Pittsburgh, PA

## Abstract

Scalable and accurate human motion tracking is expected to modernize the diagnosis and prognosis of gait pathologies, sports performance optimization, and human movement research at large. While recent advances in computer vision are promising, innovation has primarily focused on single-view approaches, which are convenient and could be applied to videos from commodity devices, such as smartphones. However, a range of biomedical applications require higher accuracies. Current multi-view tools either lack sufficient accuracy to justify the added burden of camera calibration or require higher-density multi-camera setups that discourage adoption in out-of-laboratory settings. Here, we present DeepGaitLab, an open-source framework that yields accurate three-dimensional (3D) kinematics while operating flexibly across a range of camera configurations, including only two, and foregoes the time-consuming step of inter-camera calibration. Trained on large synthetic data, DeepGaitLab overcomes the accuracy and generalizability constraints of current tools relying on real data. It outperforms both commercial and open-source alternatives and exhibits monotonic accuracy improvement with additional cameras. Unlike existing systems, its accuracy does not degrade when applied to individuals with mobility limitations. We evaluated DeepGaitLab in 80 individuals, including healthy adults, individuals recovering from anterior cruciate ligament reconstruction, individuals recovering from stroke, and ones with mild cognitive impairment, captured in two distinct environments. In addition to outperforming existing tools and demonstrating utility across three clinically distinct populations, DeepGaitLab offers the optional feature to enhance accuracy via an environment-specific fine-tuning strategy without requiring new labeled data. Together, these advancements establish DeepGaitLab as a practical and scalable platform for real-world deployment, bridging the gap between research-grade biomechanics, emerging artificial intelligence (AI) tools, and clinical impact. All the data, code, and trained models are publicly shared.

## Introduction

Scalable human motion tracking tools could reshape biomechanics research, clinical care, and athletic performance optimization. Kinematics, kinetics, and spatiotemporal gait parameters can reveal neuromuscular deficits^[1,2]^, quantify functional recovery^[3,4]^, and serve as objective biomarkers of disease progression^[5,6]^. Yet, despite decades of motion-analysis research, these measurements remain absent from routine clinical and athletic-training workflows. Traditional marker-based motion capture systems, while accurate, require dedicated infrastructure and specialized personnel, which restricts their use to research laboratories and limits the translation of research findings into practice. For example, even when kinematic or kinetic outcomes have been found to be indicative of disease-progression risk^[6,7]^, their implementation in clinical decision-making has not been possible due to a lack of tools to feasibly measure these outcomes at scale. As a result, most clinical decisions, including personalization of physical therapy, continue to rely on subjective visual assessment, rather than quantitative evidence.

Markerless motion capture offers a new pathway to overcoming these scalability barriers and enabling broader adoption of quantitative motion analysis. The last decade has been marked by a growing number of aspirational clinical testbeds across the world piloting the use of vision-based motion tracking in clinics^[8–10]^. By eliminating the need for expert-placed markers, circumventing postprocessing pipelines that require specialized training, and reducing hardware cost requirements, markerless systems could offer clinicians research-grade data and researchers clinical-scale data, closing the loop between research and clinical practice. Nevertheless, existing markerless tracking solutions^[11,12]^ have yet to optimally balance the accuracy and flexibility required to inspire widespread adoption. Single-camera methods^[13–15]^ are accessible but may not be sufficiently accurate across all applications due to depth and occlusion ambiguity. Multi-camera markerless systems offer higher accuracy^[16]^ but present practical limitations: the accuracy of open-source platforms such as OpenCap^[11]^ does not improve meaningfully with more than two cameras, while commercial systems such as Theia3D^[12]^ require dense camera arrays and proprietary software, making them difficult to deploy in under-resourced clinics and laboratories. Additionally, clinical environments prioritize ease of use, while current multi-camera tools require time-consuming calibration steps.

Despite rapid progress in computer vision, particularly in pose estimation and human mesh recovery^[13–15]^, these advances have not translated into equally rapid innovation in multi-view biomechanical analysis. The computer vision community has focused largely on single-view algorithms optimized for in-the-wild imagery^[17–19]^, prioritizing robustness to cluttered scenes and pose variability rather than the accuracies required for clinical gait assessment. Meanwhile, the biomechanics community has generally adopted these models as fixed components, instead of developing new architectures, often focusing innovation on downstream musculoskeletal modeling rather than upstream three-dimensional (3D) reconstruction. As a result, contemporary tools reflect a methodological gap: OpenCap^[11]^ integrates state-of-the-art single-view algorithms with musculoskeletal modeling pipelines but inherits the depth ambiguity and landmark sparsity of those networks, placing an upper bound on achievable accuracy as the number of cameras increases. Conversely, commercial systems such as Theia3D circumvent algorithmic limitations by relying on hand-labeled multi-view datasets with denser body landmarks, a strategy that is difficult to scale, opaque to the research community, and constrained by proprietary training corpora affordable only to industry developers. Together, these dynamics have left multi-view markerless motion capture without clear methodological innovation that could simultaneously improve accuracy, generalizability, and feasibility. Closing this gap requires models that leverage the rigor and reproducibility sought by biomechanists while drawing from advances in computer vision, particularly synthetic data generation^[20–22]^ and multi-view geometric learning^[23,24]^, to deliver clinically acceptable accuracy without relying on labor-intensive annotation and data-collection pipelines.

**Figure 1.**
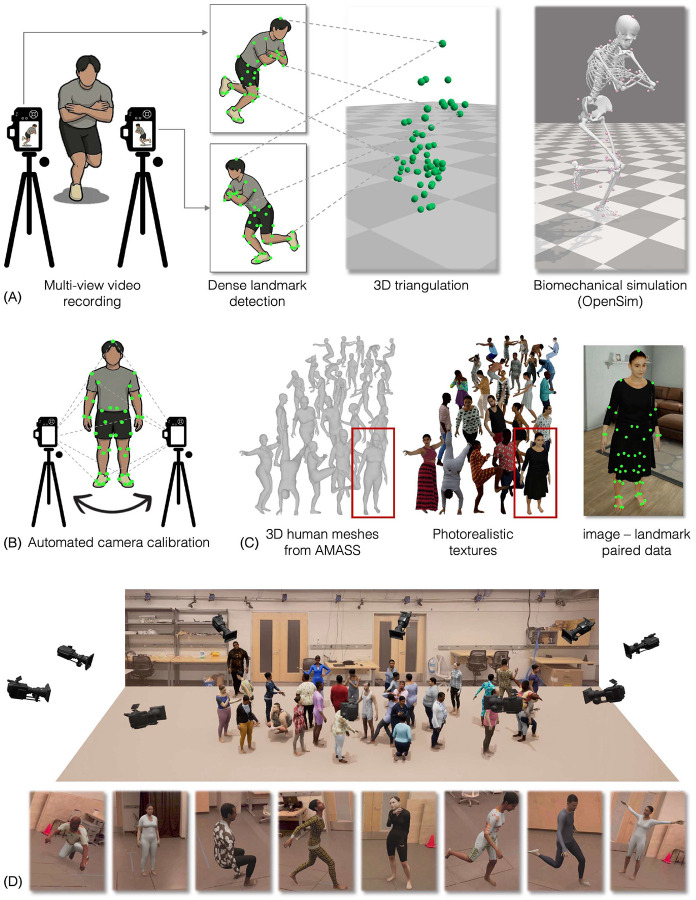
DeepGaitLab Overview. (A) DeepGaitLab estimates three-dimensional (3D) kinematics from multi-view red-green-blue (RGB) videos using a three-stage workflow: (i) multi-view video recording and dense landmark detection in two-dimensional (2D) space, (ii) triangulation of landmarks into 3D virtual markers, and (iii) biomechanical simulation in OpenSim. (B) The framework offers an automated calibration feature that estimates camera extrinsics by utilizing the subject as a dynamic-calibration object, obviating the need for physical calibration. (C) The dense landmark detection model is trained on large-scale synthetic data, wherein 271 real human bodies represented as meshed models (AMASS dataset), performing 9.3 hours of real recorded motions, are synthetically dressed and projected on diverse imagery to create a large dataset of x–y pairings used to train the landmark detection model. Here, x is the synthetic image, while y is the landmark location based on the true meshed model pose and shape. (D) Synthetic humans with photorealistic textures can also be rendered within a target laboratory’s camera configuration and background images, enabling fine-tuning of DeepGaitLab to a specific laboratory without any manual annotation. An environment-specific multi-view video dataset is generated with diverse body poses, shapes, colors, and clothing.

Here, we introduce DeepGaitLab, an open-source multi-view markerless motion capture framework designed to address this gap (Fig. 1). DeepGaitLab delivers accurate 3D kinematics across a broad range of camera configurations, from two-camera setups suitable for standard clinical settings to high-density arrays used in gait laboratories (Fig. 1A), and it does not require inter-camera spatial calibration (Fig. 1B). This flexibility allows researchers and clinicians to tailor capture protocols to their space, budget, and accuracy needs. DeepGaitLab is built using synthetic data (Fig. 1C) and outperforms existing open-source and commercial systems at matched camera counts. Unlike current open-source tools, its accuracy improves consistently with additional cameras. Moreover, DeepGaitLab maintains unbiased performance in individuals with mobility limitations, even when existing tools do not. Finally, DeepGaitLab supports environment-specific fine-tuning that leverages synthetic data matched to a site’s camera geometry and appearance to further improve accuracy without new labeled data (Fig. 1D).

## Results

### User experience: data collection and processing with DeepGaitLab

DeepGaitLab is hardware-agnostic and can be accessed through a web-based interface. It accepts data from any combination of standard red-green-blue (RGB) cameras and supports two acquisition workflows. For controlled settings where hardware synchronization via electrical triggers and multi-view calibration using checkerboards are available, users can upload synchronized videos with calibration data. This workflow is encouraged to maximize tracking accuracy when such infrastructure is available. Alternatively, for data collection in natural environments that lack centralized camera control or have time constraints, DeepGaitLab accepts independently recorded videos, offering automatic camera synchronization and calibration. Once the video data are processed, 3D virtual-marker positions are estimated through a deep learning model. Subject scaling and inverse kinematics are then performed automatically in the OpenSim software^[25,26]^ (Fig. 1). The conversion to an OpenSim model ensures that kinematics are expressed using the Grood and Suntay convention^[27]^ and facilitates downstream computation of kinetic outcomes, such as joint moments, when they are of interest. DeepGaitLab can also be adapted to interface with other biomechanical modeling platforms.

### Inverse kinematics in adults without gait impairments

**Figure 2.**
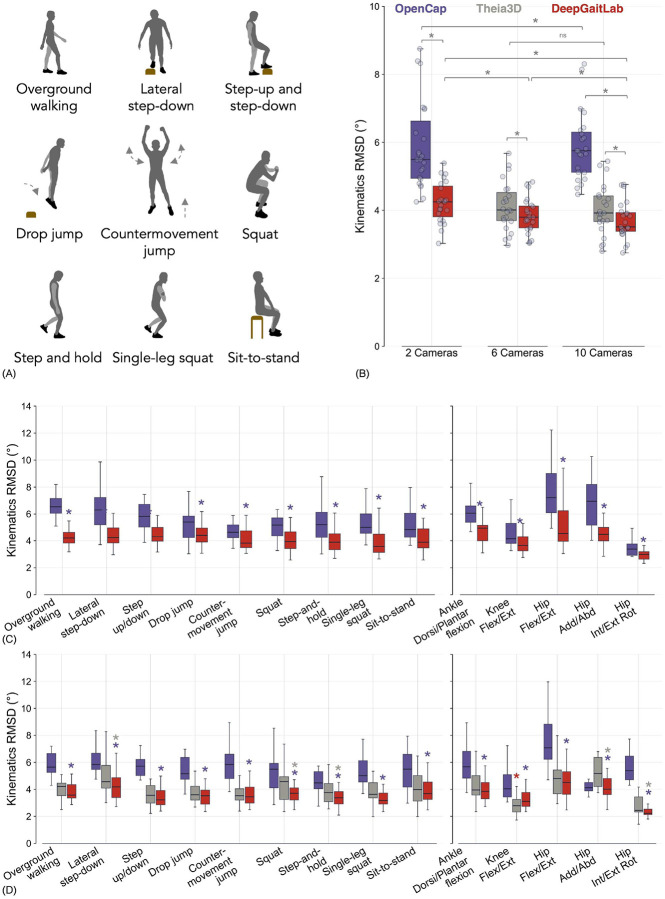
Inverse Kinematics Performance in People without Mobility Limitations. (A) Healthy participants from the I-MOVE-23 dataset performed a wide range of activities while being simultaneously recorded by 20 infrared and 10 RGB cameras for marker-based and markerless analysis, respectively. (B) DeepGaitLab achieved higher kinematic agreement (i.e., lower error) with the marker-based reference than OpenCap and Theia3D across camera configurations (Comparison 1). DeepGaitLab’s performance improves with more cameras, while that of the other two tools does not (Comparison 2). (C) In the minimal, two-camera configuration, DeepGaitLab outperforms OpenCap across activities and degrees of freedom. (D) In denser camera setups, DeepGaitLab outperforms both OpenCap and Theia3D, particularly in non-locomotion activities. In each box plot, the horizontal line indicates the median, the box spans the interquartile range (25th to 75th percentiles), and the whiskers represent 1.5 times the interquartile range.

We first benchmarked DeepGaitLab against OpenCap^[11]^ and Theia3D^[12]^, the leading open-source and commercial markerless solutions, using the I-MOVE-23 dataset^[16]^. In this dataset, twenty-three participants without any gait impairments performed overground walking and eight physical therapy exercises while being simultaneously recorded with marker-based and markerless systems (Fig. 2A). DeepGaitLab consistently achieved higher agreement with the marker-based reference than the two alternatives, across all camera configurations (Fig. 2B; Comparison 1). In the two-camera setting, DeepGaitLab outperformed OpenCap, demonstrating a significantly lower root mean square difference (RMSD) from marker-based analysis, across all tasks and lower-limb degrees of freedom (4.2 ± 0.6° vs. 5.9 ± 1.3°, p<0.0001) (Fig. 2C). DeepGaitLab yielded lower RMSDs than Theia3D in both the six-camera (3.8° ± 0.5° vs. 4.1° ± 0.7°; p = 0.0003) and ten-camera configurations (3.7° ± 0.6° vs. 4.0° ± 0.7°; p < 0.0001). Particularly, DeepGaitLab showed higher agreement with marker-based analysis than Theia3D in the lateral step-down, squat, and step-and-hold tasks and non-sagittal degrees of freedom when using ten cameras (Fig. 2D). These camera densities were selected because two is the minimum number with which OpenCap and DeepGaitLab operate, six is the minimum for Theia3D, and ten is the maximum number of RGB video data we collected.

Next, we assessed the effect of camera density on each of the three tools. For OpenCap, increasing the number of cameras from two to ten did not result in a significant reduction in RMSD (5.9 ± 1.3° for two vs. 5.9 ± 1.0 for ten cameras, p = 0.7020) (Fig. 2B; Comparison 2). In contrast, DeepGaitLab exhibited a significant reduction in RMSD as the number of cameras gradually increased from two to ten (4.2 ± 0.6° for two vs. 3.8 ± 0.5° for six cameras, p<0.0001; 3.8 ± 0.5° for six vs. 3.7 ± 0.5 for ten cameras, p = 0.0067) (Fig. 2B; Comparison 2). For Theia3D, increasing the number of cameras from six to ten did not result in a significant reduction in RMSD (4.1 ± 0.7° for six vs. 4.0 ± 0.7° for ten cameras, p = 0.0977) (Fig. 2B; Comparison 2).

### Inverse kinematics in individuals with ACL reconstruction

**Figure 3.**
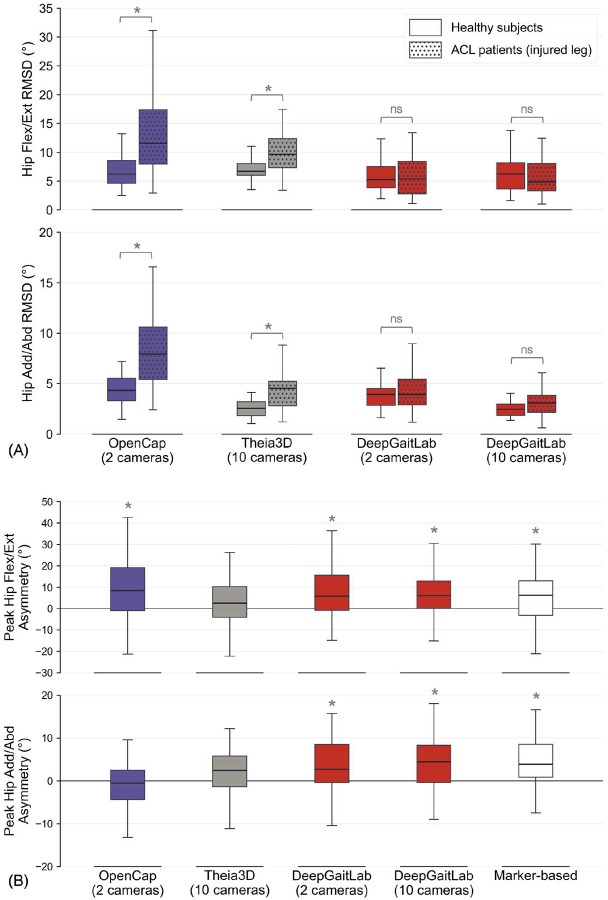
Inverse Kinematics Performance in People Following ACL Reconstruction. (A) Unlike other markerless tools, which showed lower agreement with marker-based tracking for people with ACLR relative to those without mobility limitations, DeepGaitLab maintained consistent accuracy during the recorded lateral step-down task. (B) DeepGaitLab successfully captured the interlimb asymmetries in hip flexion/extension and adduction/abduction present in the marker-based reference. In contrast, OpenCap did not capture hip adduction/abduction asymmetry, and Theia3D captured neither asymmetry. In each box plot, the horizontal line indicates the median, the box spans the interquartile range (25th to 75th percentiles), and the whiskers represent 1.5 times the interquartile range.

Computer vision tools are often trained and tested using data from people without major gait pathologies, making it unclear whether these methods generalize to people with gait impairments and thereby support clinical diagnosis. We therefore tested DeepGaitLab’s performance in thirty-eight people with anterior cruciate reconstruction (ACLR), while they performed a lateral step-down task, which is a common functional exercise used to assess rehabilitation progress in this patient population. The performance of both OpenCap and Theia3D depended on the interaction between mobility status (healthy vs. ACLR) and degree of freedom (p < 0.0001 for OpenCap and p = 0.0002 for Theia3D) (Fig. 3A). ACLR participants showed higher RMSDs compared to those without mobility limitations, for hip flexion/extension (13.4 ± 7.9° vs. 7.3 ± 4.3°, p = 0.0001 for OpenCap and 9.8 ± 3.9° vs. 7.2 ± 2.6°, p = 0.0217 for Theia3D) and for hip adduction/abduction (8.3 ± 3.6° vs. 4.4 ± 1.5°, p<0.0001 for OpenCap and 4.4 ± 2.0 vs. 2.5 ± 0.9°, p = 0.0001 for Theia3D) (Fig. 3A). In contrast, DeepGaitLab maintained consistent performance in the two groups, showing no significant differences between people with ACLR and those without mobility limitations, in either the two-camera (p = 0.1208) or ten-camera (p = 0.3151) setups. DeepGaitLab also showed no significant interaction between mobility status and kinematic degree of freedom, for both two-camera (p = 0.0892) and ten-camera (p = 0.1413) configurations (Fig. 3A).

Finally, we tested whether each of the three tools could be used to detect an interlimb asymmetry in people three months after ACLR surgery, which is detectable with marker-based motion capture and bears clinical significance^[28]^. During the lateral step-down exercise, marker-based motion capture identified a significant peak hip flexion/extension asymmetry (5.9 ± 11.7°, p = 0.0032). This asymmetry was successfully detected by OpenCap (10.0 ± 14.9°, p = 0.0003) but not by Theia3D (3.2 ± 10.6°, p = 0.0661). With DeepGaitLab, the asymmetry was detectable in both its two-camera (7.1 ± 11.5°, p = 0.0009) and ten-camera (6.7 ± 10.4°, p = 0.0005) configurations (Fig. 3B). Similarly, marker-based analysis revealed a significant peak hip adduction/abduction asymmetry (4.2 ± 5.8°, p = 0.0002). Neither Theia3D (1.8 ± 6.4°, p = 0.0748) nor OpenCap (−1.4 ± 5.5°, p = 0.1718) detected this asymmetry. In contrast, the asymmetry was successfully captured by DeepGaitLab in both its two-camera (3.0 ± 6.4°, p = 0.0101) and ten-camera (3.6 ± 6.5°, p = 0.0028) configurations (Fig. 3B).

### Spatiotemporal asymmetry in people with mild cognitive impairment or stroke

**Figure 4.**
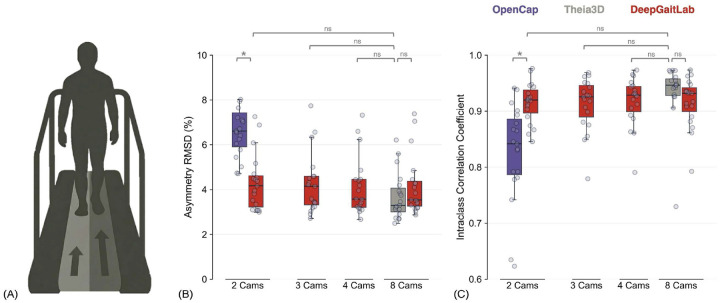
Spatiotemporal Asymmetry Performance in People with Mild Cognitive Impairment or Stroke. (A) Participants walked on a split-belt treadmill with asymmetric belt speeds while recorded by markerless and marker-based systems. (B) In the two-camera configuration, DeepGaitLab achieved higher step-length asymmetry agreement with marker-based analysis than OpenCap. It matched Theia3D’s eight-camera performance with only two cameras. (C) DeepGaitLab demonstrated a higher correlation with marker-based analysis than OpenCap and a comparable correlation to Theia3D with a significantly smaller number of cameras. In each box plot, the horizontal line indicates the median, the box spans the interquartile range (25th to 75th percentiles), and the whiskers extend to the furthest data points within the 1.5 × interquartile range of the quartiles.

After assessing DeepGaitLab’s performance in people with orthopedic injuries, such as ACLR, we evaluated its performance in people with neurological conditions. Data were collected from eight individuals with mild cognitive impairment (MCI) and eleven stroke survivors performing treadmill walking with split-belt perturbations (Fig. 4A), which enabled evaluation of recovery from perturbations. We focused on step-length asymmetry as the outcome of interest, given its relevance in these two populations (Fig. 4B and C). In the two-camera configuration, DeepGaitLab outperformed OpenCap in asymmetry agreement with marker-based motion capture, achieving significantly lower RMSDs (4.32 ± 1.27% vs. 6.83 ± 1.57%, p<0.0001) and higher intraclass correlation coefficients (ICC; 0.91 ± 0.04 vs. 0.83 ± 0.09, p<0.0001). When tested with eight cameras, DeepGaitLab performed comparably with Theia3D in both RMSD (4.16 ± 1.33% vs. 3.69 ± 1.04%, p = 0.0141) and ICC (0.92 ± 0.04 vs. 0.92 ± 0.10, p = 0.0323); however, neither comparison remained significant after Holm–Bonferroni correction for the multiple pairwise tests (adjusted p = 0.2115 and 0.2324, respectively). Importantly, DeepGaitLab showed comparable agreement to marker-based analysis even with a lower number of cameras than Theia3D (two cameras vs. eight cameras) in both RMSD (4.32 ± 1.27% vs. 3.69 ± 1.04%, p = 0.0062) and ICC (0.91 ± 0.04 vs. 0.92 ± 0.10, p = 0.0258); these differences also did not remain significant after Holm–Bonferroni correction (adjusted p = 0.0618 and 0.2324, respectively).

### Inverse dynamics

**Figure 5.**
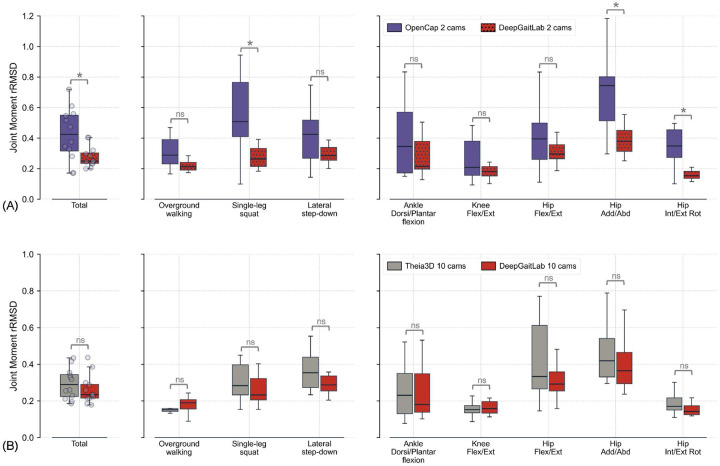
Inverse Dynamics Performance in People without Mobility Limitations. (A) Using the two-camera configuration, DeepGaitLab outperformed OpenCap, particularly during single-leg squats and in non-sagittal degrees of freedom. (B) Under the ten-camera setting, DeepGaitLab and Theia3D performed similarly across activities and degrees of freedom. In each box plot, the horizontal line indicates the median, the box spans the interquartile range (25th to 75th percentiles), and the whiskers represent 1.5 times the interquartile range.

Next, we compared DeepGaitLab’s performance in estimating lower-extremity joint moments. We used the relative root mean square difference (rRMSD) as the primary evaluation metric. This metric is defined as the RMSD normalized by the range of the marker-based reference moment, yielding a unitless measure of relative error that ranges from 0 to 1. When using two cameras (Fig. 5A), DeepGaitLab yielded a lower rRMSD from the marker-based inverse dynamics than OpenCap (0.27 ± 0.07 vs. 0.42 ± 0.17, p<0.0001). The largest performance gap was observed during the single-leg squat activity (0.30 ± 0.12 vs. 0.54 ± 0.26, p<0.0001) and in non-sagittal degrees of freedom (0.41 ± 0.14 vs. 0.69 ± 0.27, p<0.0001 for hip adduction/abduction moment and 0.17 ± 0.05 vs. 0.33 ± 0.15, p<0.0001 for hip internal/external rotation). When using the denser camera setup, the performances of DeepGaitLab and Theia3D were comparable (Fig. 5B). Notably, however, DeepGaitLab reached rRMSDs comparable to those of Theia3D even with only two cameras, during single-leg squat (0.30 ± 0.12 for DeepGaitLab with two cameras vs. 0.34 ± 0.17 for Theia3D with ten, p = 0.0909) and lateral step-down (0.30 ± 0.08 for DeepGaitLab with two cameras vs. 0.37 ± 0.12 for Theia3D with ten, p = 0.1843) but not during walking (0.22 ± 0.03 for DeepGaitLab with two cameras vs. 0.16 ± 0.03 for Theia3D with ten, p < 0.0001). For overground walking, DeepGaitLab and Theia3D performed comparably only when using the same camera density. Unlike stationary exercises such as the lateral step-down, overground walking is typically performed across a specified runway, with subjects not maintaining the same distance relative to a camera and occasionally exiting the field of view of both cameras during the two-camera setup. The resulting need for trajectory interpolation when the subject is outside the field of view therefore leads to reduced accuracy compared to higher-density configurations that provide coverage over more of the motion capture space.

### Inverse kinematics evaluation with the optional autocalibration feature

**Figure 6.**
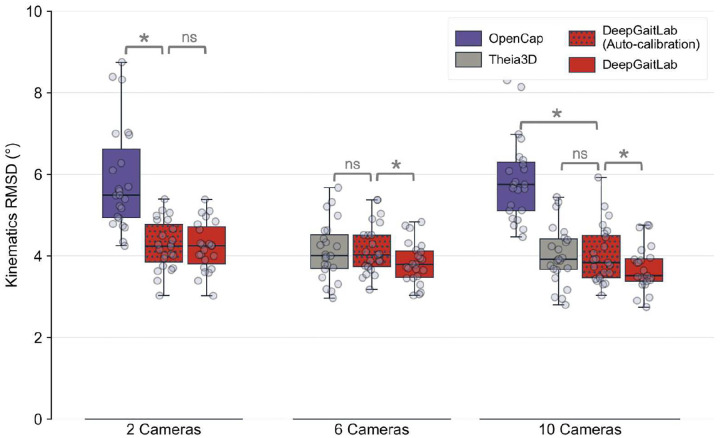
Effect of Autocalibration on Inverse Kinematics Performance. DeepGaitLab with automated calibration outperformed OpenCap with manual calibration and matched Theia3D with manual calibration. In each box plot, the horizontal line indicates the median, the box spans the interquartile range (25th to 75th percentiles), and the whiskers represent 1.5 times the interquartile range.

Given the resource-demanding step of temporal synchronization and spatial calibration, we imparted DeepGaitLab with automated inter-camera synchronization and calibration features, which should facilitate uptake in clinical settings. In the two-camera configuration, DeepGaitLab with automated calibration achieved similar RMSDs to DeepGaitLab with ground-truth, checkerboard-informed, calibration (4.3 ± 0.6° vs. 4.2 ± 0.6°, p = 0.5054) (Fig. 6). DeepGaitLab with automated calibration outperformed OpenCap with ground-truth, checkerboard-informed calibration (4.3 ± 0.6° vs. 5.9 ± 1.3°, p<0.0001). In the denser camera configurations, DeepGaitLab with automated calibration performed similarly to Theia3D with ground-truth, checkerboard-informed, calibration, in both the six-camera (4.2 ± 0.6° vs. 4.1 ± 0.7°, p = 0.2934) and ten-camera (4.0 ± 0.7° vs. 4.0 ± 0.7°, p = 0.8586) settings. Relative to DeepGaitLab with physical, checkerboard-informed, calibration, automated calibration introduced a modest performance penalty at higher camera counts (4.2 ± 0.6° vs. 3.8 ± 0.5°, p<0.0001 for six cameras; 4.0 ± 0.7° vs. 3.7 ± 0.6°, p < 0.0001 for ten cameras).

### Inverse kinematics with the optional environment-specific fine-tuning feature

Given that research laboratories typically maintain static camera configurations and consistent lighting environments, we also developed laboratory-specific models tailored to these fixed conditions. We implemented a domain adaptation framework to generate synthetic training data that replicate the target laboratory’s visual characteristics and fine-tuned the pretrained landmark detection model on those data. When evaluated on the I-MOVE-23 dataset that includes various physical therapy exercises, such as squatting and sit-to-stand, this feature did not improve performance (3.7 ± 0.7° vs. 3.7 ± 0.6°, p = 0.6784). Its benefits were more salient during dynamic activities, such as overground walking (3.4 ± 0.7° vs. 3.8 ± 0.6°, p = 0.0250) across degrees of freedom. The largest gain was found in knee flexion/extension (3.0 ± 0.7° vs. 3.6 ± 0.6°, p = 0.0004) and ankle dorsiflexion/extension (3.9 ± 0.8° vs. 4.6 ± 1.2°, p = 0.0038). Given that marker-based motion capture has been shown to have errors of 2° to 4° for the sagittal plane^[29]^ and 5° to 8° for non-sagittal plane kinematics^[30]^, this flooring effect may be due to the markerless tool approaching the marker-based motion tracking error margins.

## Discussion

This study demonstrates that accurate markerless motion tracking can be achieved with lightweight multi-view camera setups and without the need for large human-annotated datasets. Across healthy adults, individuals post-ACLR, individuals with mild cognitive impairment, and patients recovering from stroke, the newly introduced DeepGaitLab outperformed existing tools. This performance advantage is due to a few key design choices. First, predicting dense surface landmarks rather than sparse joint centers provides the necessary geometric constraints to resolve segment orientations more accurately, addressing a limitation of prior, keypoint-based approaches. Since sparse joint centers leave rotational degrees of freedom ambiguous, these approaches often rely on statistical motion priors to infer rotations^[14,31]^ or use deep learning models to map sparse joint centers to dense virtual markers^[11]^, an inherently under-constrained problem. By explicitly constraining degrees of freedom with observed image evidence, DeepGaitLab mitigates bias toward motions represented in the training data, thereby preserving generalization. Second, DeepGaitLab is trained on large-scale synthetic data derived from the BEDLAM dataset^[20]^. Supervision with synthetic data enables dense, anatomically consistent labels across a wide range of poses and body shapes, which is infeasible to obtain manually. This strategy avoids the prohibitive requirement of hand-labeling multi-camera datasets, a constraint that fundamentally limits current pipelines, and supports generalization across camera placements, motion types, and people. Third, the monotonic improvement in accuracy with additional cameras, which further distinguishes DeepGaitLab from existing systems, is driven by our dense landmark configuration and a transfer-learning strategy that was initialized with features learned from large-scale real-world images and then fine-tuned on synthetic data with dense annotations. As a result, DeepGaitLab’s dense landmark prediction resolves the ambiguity of segment orientation inherent in approaches that rely on sparse keypoints. The accuracy of sparse joint-center-based methods such as OpenCap plateaus because of their reliance on keypoint estimators^[32,33]^ that predict joint centers. However, even perfectly triangulated joint centers are geometrically insufficient to define a 3D segment orientation. Consequently, while adding cameras improves the 3D position of these points, it does not constrain the segment orientation, leading to the observed performance plateau. Camera count alone, therefore, does not determine accuracy. Rather, it is the combination of encoded representations, the quality of the training data, and the multi-view fusion strategy that governs whether additional views translate into meaningful performance gains.

An advantage of vision-based methods such as DeepGaitLab over closed, proprietary, marker-based systems is that improvements in computer vision algorithms can directly translate into improved robustness and accuracy. As accuracy improves, however, advances will be increasingly difficult to measure due to the lack of a true “ground truth” limiting the field. Marker-based systems have long been treated as the “gold standard,” but vision-based methods could surpass them. At that point, new definitions of accuracy will be needed. Clinical evaluation is a possible new metric for evaluation, wherein the performance of such systems is judged on ability to support the diagnosis of disease or impairment.

Prior work has reported marker-based motion tracking to have errors of 2° to 8° across lower-limb joints^[29,30]^, a range that aligns with the agreement between DeepGaitLab and marker-based systems. This limitation is well-documented in classical studies using intracortical bone pins. Reinschmidt et al.^[30]^ and Lafortune et al.^[34]^ demonstrated that while skin markers track sagittal plane motion reliably, soft tissue artifacts introduce substantial errors in secondary planes, with knee rotations often deviating by 5° to 8° during running. Similarly, Nester et al.^[35]^ reported that surface markers fail to capture the complex mechanics of the foot, yielding errors of up to 6° relative to bone-mounted references. Recent validations using biplanar videoradiography and radiostereometric analysis support these historical findings. Benoit et al.^[36]^ and Miranda et al.^[29]^ confirmed that during high-intensity activities such as jump cutting, skin deformation can decouple markers from the underlying bone, causing rotational errors that exceed 10° in the transverse plane. These studies underscore a limitation of current validation paradigms used for emerging markerless tracking approaches. DeepGaitLab’s marginal gains observed with environment-specific fine-tuning may reflect proximity to the marker-based performance floor. Further progress in markerless systems will therefore require higher-fidelity references, such as biplane radiography, to disambiguate whether a flooring effect in performance should be interpreted as such or whether it is due to a suboptimal reference. The necessity for new references was recently highlighted by a study showing that inertial tracking and marker-based motion tracking exhibit similar agreement to biplane radiography^[37]^. Ionizing radiation and limited field of view, however, continue to make biplane radiography a challenging gold standard, despite its high accuracy.

In the context of inverse dynamics, even minor kinematic errors are amplified, as joint moments are highly sensitive to the calculated moment arm of external forces. Joint moments are second-order quantities that amplify upstream errors through numerical differentiation and dynamic coupling with ground reaction forces. Marker-based inverse dynamics exhibit significant sensitivity to joint-center mislocation and segment orientation errors, with reported uncertainties of 10% to 20% for sagittal-plane moments^[38–40]^ and substantially larger discrepancies for frontal and transverse plane moments^[41]^. Prior work^[42][43]^ has further revealed that even small kinematic errors can lead to disproportionately large errors in joint moments, particularly at the hip flexion moment. Markerless systems, including DeepGaitLab, can now approach the accuracy of marker-based kinematics^[29,30]^ but still lag in kinetics. A likely contributing factor is the lack of physical anchoring to palpated anatomical landmarks. While marker-based systems define joint centers relative to bony prominences identified by trained researchers or clinicians, markerless systems must regress these deep internal anatomical features from the external surface envelope, which may include clothing. A minor bias in estimating the subject’s joint centers may not substantially affect segment orientations and rotational joint kinematics, but it can detrimentally corrupt the estimation of the moment arm. This shortfall could be addressed through multiple directions. One approach is to integrate physics-informed constraints that enforce dynamic consistency during the tracking phase, or to use personalized bone geometry priors to better constrain the internal skeleton within the image evidence. Alternatively, improvements in training data may allow models to implicitly learn these internal anatomical relationships without the need for explicit physics-based constraints. Specifically, synthetic datasets that accurately model soft-tissue dynamics hold untapped promise.

As a result, despite its strengths, DeepGaitLab has room for improvement. As synthetic generation pipelines (e.g., BEDLAM^[20,21]^, MammaSyn^[24]^) become increasingly more photorealistic and capable of modeling person–person and person–scene interactions, as well as soft-tissue motion, they may enable better personalized models. Second, incorporating learned uncertainty, visibility prediction, or contact reasoning could further strengthen the system’s robustness under occlusion or constrained camera placement. Third, DeepGaitLab, like most current markerless systems, operates as a two-stage pipeline that detects virtual markers using computer vision and subsequently maps them to a generic, linearly scaled biomechanical model. This approach overlooks subject-specific anatomical variations not captured by the current pipeline’s linear scaling strategy. While more expressive body models such as SMPL^[44,45]^ can represent shape variation beyond simple scaling, incorporating such representations to capture subject-specific bone geometries and soft-tissue distributions remains an untapped direction for future work. Fourth, similar to other markerless systems, DeepGaitLab processes motion as a kinematic sequence without encoding underlying Newtonian laws that govern human movement. While this results in accurate kinematic capture, the resulting motions may lack dynamic consistency when coupled with physics-based simulation. Future integration of physics-informed loss functions within the training loop could constrain predictions to be not only visually plausible but also dynamically plausible. Finally, the focus of this study was the evaluation of lower-extremity biomechanics, and it is still unclear whether DeepGaitLab outperforms existing tools in upper-extremity biomechanics, which remains a focus of future studies.

Markerless tracking, beyond DeepGaitLab, has other limitations that remain to be addressed. Video capture generates large data that pose challenges given that storage is expensive. When matching the same data-collection frequency (e.g., 120 Hz), two minutes of walking data will result in 40MB for marker-based motion tracking and 3GB for two-camera markerless tracking. The current methods also do not operate in real time. Unlike marker-based motion tracking, however, markerless tracking requires minimal user input. Many biomedical applications can tolerate the lack of real-time operability. Akin to bloodwork and many other clinical tests that require processing time, we do not foresee a time lag in processing of motion data to be prohibitive of clinical translation. Currently, DeepGaitLab takes approximately 15 minutes to process two minutes of data from two cameras on a consumer-grade GPU (RTX 5090). This performance is comparable to commercial markerless solutions like Theia3D, which require offline batch processing. Future work, however, could focus on computational efficiency and real-time operability.

While algorithmic advances will continue to propel markerless tracking toward replacing marker-based analysis, DeepGaitLab already provides an exciting and adaptable foundation, without the accuracy, scalability, and transparency tradeoffs that may have limited prior tools. By demonstrating that research-grade motion analysis can be achieved with consumer-grade RGB camera setups and synthetic supervision, DeepGaitLab redefines what is feasible outside of specialized laboratories. This democratization can reshape the diagnosis and prognosis of mobility-limiting conditions, athletic performance optimization, and movement sciences at large.

## Methods

### Overview of the DeepGaitLab framework

DeepGaitLab is a multistage framework that estimates 3D human body kinematics from multi-view RGB videos (Fig. 1A). The system processes video inputs through three sequential stages: (1) 2D dense anatomical landmark estimation for each camera view, (2) triangulation of 2D anatomical landmarks into 3D space, and (3) biomechanically constrained inverse kinematics using the OpenSim software. The dense landmark detection stage follows multiple steps to robustly identify and track subjects across video frames. First, the YOLOv8 object-detection model^[46]^ is applied to the initial frame of each video to identify all the individuals present in the scene. The user then selects the target subject to be tracked, enabling DeepGaitLab to focus solely on that individual of interest when more than one person is in the field of view. Next, the SAMURAI tracking algorithm^[47]^ propagates the selected bounding box through subsequent frames and generates a segmentation mask for the subject in each view, effectively isolating the person from background clutter. Finally, the cropped image and corresponding segmentation mask are passed as inputs into a dense landmark detection network, which estimates the pixel coordinates of each landmark in the view.

In this framework, landmarks are defined as customizable points on a body mesh that correspond to salient anatomical locations, or virtual markers akin to physical markers in marker-based analysis. We detect a high number of surface landmarks, as opposed to commonly used keypoints (i.e., joint centers) in computer vision, because this dense representation helps with occlusion and preserves more information from a single video to help reconstruct body-segment orientations, resulting in higher motion tracking accuracy and higher gains with more cameras. The number of surface landmarks is customizable; we define our 57-landmark configuration as “dense” to contrast the sparse 17-keypoint output of standard computer vision models. In defining these landmarks, we use anatomical marker protocols typical of marker-based motion capture. We selected an even higher density after finding that standard marker-based configurations were vulnerable to rotational noise when applied to markerless estimation, requiring additional points to robustly constrain body segment orientation. Following this per-view detection, the 2D landmark coordinates from all the videos are triangulated into 3D world coordinates using a linear least-squares approach that leverages the known camera calibration parameters. The trajectory of 3D landmarks then serves as input for the OpenSim software to scale the subject and compute kinematics.

### Dense landmark prediction network and training on synthetic data

**Figure 8.**
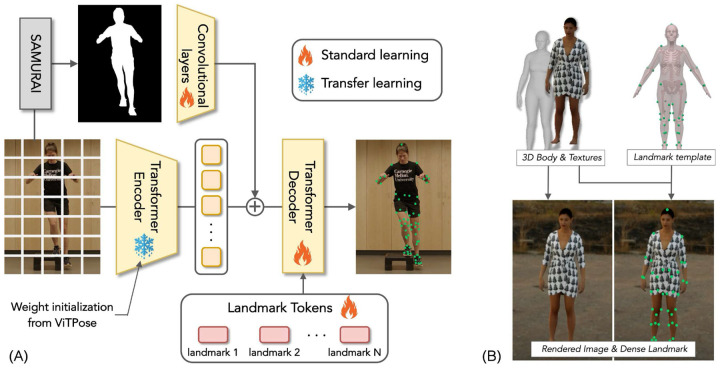
Dense Landmark Detection Network Architecture. (A) We use SAMURAI^[47]^, a video segmentation model, to extract segmentation masks from RGB images. A Transformer encoder extracts features from images, while masks are processed by convolutional layers. The combined features are passed to a Transformer decoder that predicts dense 2D anatomical landmark coordinates using learnable per-marker tokens. The melting snowflake icon indicates that the weights of Transformer encoder were initialized with ViTPose^[55,56]^, a keypoint detection model trained on large-scale image datasets^[50–52]^, followed by fine-tuning on synthetic data (transfer learning). The flame icon indicates modules trained from scratch (standard learning). (B) Synthetic data provide ground-truth 3D body geometry and realistically textured images, where paired dense landmark annotations are derived from the underlying geometry and a user-configurable landmark template. These synthetic data are used to fine-tune the Transformer encoder and train all other modules via standard learning.

We trained a dense landmark-detection model designed to estimate customized anatomical landmarks directly from each frame of a video. The detection architecture is composed of multiple Transformer networks^[48]^, using a Transformer encoder^[49]^ to encode the input RGB image features and a Transformer decoder to predict the specific 2D coordinates of each landmark^[24]^ (Fig. 8A). A segmentation mask extracted using SAMURAI^[47]^ is added to the image features. One approach to training such a network would be to manually annotate features on the body in large image datasets such as COCO^[50]^, MPII^[51]^ and AI Challenger^[52]^ datasets. This approach, however, is resource-intensive and limited by variability among human annotators. Instead, we trained our system on a synthetic dataset, BEDLAM^[20]^, that has high-quality human-body annotations and photorealistic synthetic images (Fig. 8B). BEDLAM provides ground-truth 3D body geometry and motion, with body shapes and poses derived from the AMASS dataset^[53]^; we use a total of 9.3 hours of marker-based motion capture data from 271 individuals^[53]^ (Fig. 1A). Human body geometry is represented using a 3D parametric meshed model^[44,45,54]^. In addition, the dataset also provides paired synthetic images rendered with the realistic textures and clothes using an advanced game engine (Unreal Engine; Epic Games, Cary, NC). We defined a landmark template with 57 anatomical landmarks, from which we obtained the ground-truth 2D coordinates of the landmarks corresponding to the synthetic images. Training the detection network solely on the synthetic data could have overfit the model to the synthetic images, risking loss of generalization to new environments. To mitigate this risk, we employed a transfer-learning strategy. Specifically, we initialized the Transformer encoder with weights from an off-the-shelf sparse joint-center detection network (ViTPose^[55,56]^), which was pre-trained on large-scale real image data^[50–52]^. Subsequently, we trained a Transformer decoder on the BEDLAM dataset, while fine-tuning the Transformer encoder using a slow learning rate (Fig. 8).

### Triangulation and biomechanical simulation

The dense 2D landmarks predicted from each camera view were triangulated into 3D space using a linear least-squares solver^[57]^, generating a dense point cloud of virtual markers (Fig. 1A). To ensure temporal consistency and remove high-frequency noise inherent in frame-by-frame detection, we applied a low-pass filter to the 3D marker trajectories^[58,59]^. Subsequently, we estimated 3D joint kinematics using the OpenSim software^[25,26]^ (Fig. 1A). We employed the full-body musculoskeletal model developed by Rajagopal et al.^[60]^, scaling the generic model to each participant’s anthropometry based on the reconstructed marker positions from a static neutral pose. Finally, kinematics were computed using the Inverse Kinematics solver^[25]^, which minimizes the weighted squared distance between the experimental virtual markers from the meshed model and the corresponding OpenSim model markers. Instead of using conventional marker sets, such as the Rizzoli one^[61]^, we defined a new one with higher marker density.

### Human-as-checkerboard autocalibration

DeepGaitLab includes an optional automated camera calibration feature that eliminates the need for traditional checkerboard-based calibration procedures. This approach leverages the dense anatomical landmarks detected on the human body as calibration targets, treating the subject walking around the capture volume as a dynamic calibration object. The calibration pipeline estimates the relative position and orientation (i.e., extrinsics) of each camera by analyzing corresponding landmark detections across multiple camera views^[62]^. Here, we assume that the camera resolution and focal length (i.e., intrinsics) are known, as they can be obtained from manufacturers. For each pair of cameras, we first identify landmarks that are confidently detected in both views and use these correspondences to estimate the relative camera pose through geometric fitting. When more than two cameras are available, the pipeline enforces geometric consistency across all cameras, which provides additional constraints to resolve the relative distances between cameras^[62]^. The camera poses are then jointly refined using bundle adjustment^[63,64]^, which minimizes the reprojection error of all the detected landmarks across all camera pairs simultaneously. A key advantage of DeepGaitLab’s dense landmark detection is that it provides a high number of surface landmarks, which may add additional geometric constraints to improve calibration. Finally, because geometric calibration from image correspondences cannot determine the absolute scale, we recover the metric scale by triangulating the subject’s head and heel landmarks during a static pose and scaling the camera configuration to match the subject’s known height.

### Lab-specific fine-tuning

Recognizing that camera configurations and background environments remain largely static in dedicated gait laboratories, we implemented an environment-specific adaptation strategy to maximize tracking accuracy. We employed the BEDLAM rendering pipeline^[20]^ to generate a lab-specific synthetic dataset by projecting the synthetic human body on each camera’s static background image. As a result, we generated 2.8 million images, amounting to 150 minutes of motion data, tailored to our specific laboratory environment: a 1000-sqft, 20 ft by 50 ft space (Fig. 1B and 1C). Using the generated data, we subsequently fine-tuned the dense landmark detection network. As the dataset is composed of multi-view images, we introduced an additional multi-view aggregation module that aggregates encoded image features across all the calibrated cameras and propagates the fused information back to each view. This mechanism provides 3D context, enabling each camera’s dense landmark detector to leverage information from complementary views. This multi-view aggregation network was trained on the lab-specific data, while the other modules were initialized with the generic model’s weights and fine-tuned with a smaller learning rate.

### Data processing and standardization

To ensure a fair comparison across systems, given the differences in underlying biomechanical models, we standardized kinematics and kinetics estimation procedures. For inverse kinematics, potential bias arising from coordinate system mismatches between the markerless approaches and the marker-based reference was addressed using static-pose transformations. We computed transformations between each system and the reference using the middle two seconds of the static neutral pose and applied these transformations to the dynamic trials, ensuring that the comparisons focused on the systems’ abilities to track motion rather than differences in model definitions. For inverse dynamics, where joint moments are highly sensitive to model definitions, we standardized the musculoskeletal model across all systems. While DeepGaitLab natively outputs OpenSim-compatible kinematics, Theia3D outputs were converted to an OpenSim-compatible format following a previously published procedure^[65]^. This allowed all systems to be evaluated using the same Rajagopal model^[60]^.

### Validation in adults without mobility limitations

To benchmark DeepGaitLab against existing markerless motion tracking solutions in adults without gait impairment, we used the publicly available I-MOVE-23 dataset^[16]^. This dataset includes 23 healthy participants (10 females, 13 males; mean age = 36 ± 11 years, height = 1.73 ± 0.09 m, weight = 77.2 ± 18.6 kg), performing overground walking at a self-selected speed and eight lower-extremity functional exercises. After obtaining approval from the Institutional Review Board of Carnegie Mellon University (STUDY2022_00000178) and informed consent, participants were simultaneously recorded with 20 infrared and 10 RGB cameras at 100 Hz in a controlled laboratory setting. The marker-based system consisted of 8 OptiTrack Prime x41 (4.1MP resolution) and 12 OptiTrack Prime x22 (2.2MP resolution) cameras (OptiTrack, Corvallis, OR), while the markerless one consisted of 10 RGB OptiTrack Prime Color cameras with a resolution of 1920 x 1080 (OptiTrack, Corvallis, OR).

**Figure 9.**
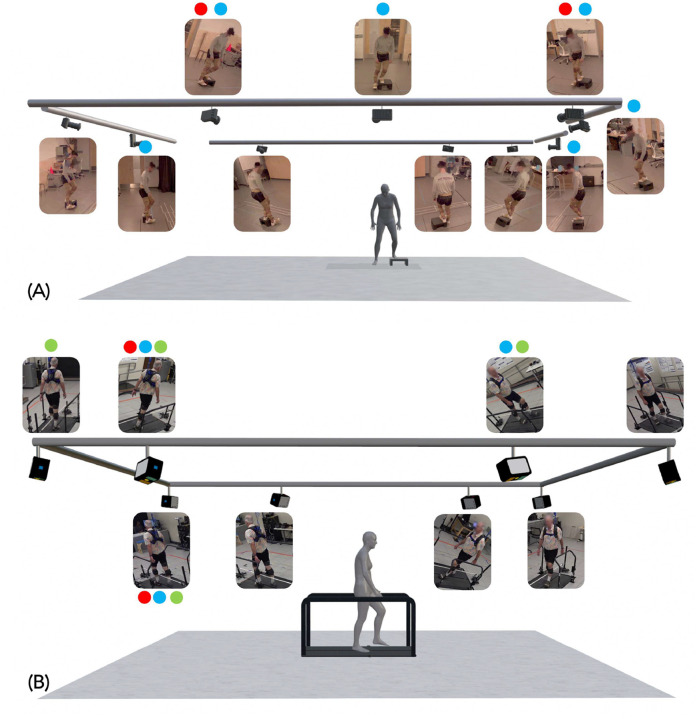
Camera Configurations for Validation Experiments. (A) The setup used for the healthy adult and ACL reconstruction validation studies included 20 infrared (IR) and 10 red-green-blue (RGB) cameras. Only the RGB camera locations are shown here. The colored indicators denote the specific subsamples used for analysis: red indicates the minimal two-camera setup, blue indicates the six-camera setup, and all cameras combined constitute the ten-camera setup. (B) The treadmill setup used for the mild cognitive impairment and stroke validation studies, consisting of 8 RGB cameras and 14 IR cameras surrounding the split-belt treadmill. Red indicates the two-camera setup, blue indicates the three-camera setup, green indicates the four-camera setup, and all the cameras constitute the eight-camera setup.

Using the same camera setups, we compared DeepGaitLab against OpenCap^[11]^ and Theia3D^[12]^, the most widely adopted open-source and commercial markerless tracking tools in biomechanics applications. For OpenCap, which requires a minimum of two cameras, we evaluated configurations using two and ten cameras, with camera pairs positioned at approximately 45° relative to the subject whenever possible. For Theia3D, which requires a minimum of six cameras, we evaluated both six- and ten-camera configurations. DeepGaitLab was evaluated across two-, six-, and ten-camera configurations to enable direct comparison with the other tools at matched camera densities. The sparser setups used a subsample of cameras from the ten-camera capture (Fig. 9A). Specifically, the two-camera configuration utilized cameras positioned at approximately 45° relative to the subject, while the six-camera configuration added four peripheral views to improve coverage (Fig. 9A, colored indicators). Additionally, we evaluated DeepGaitLab’s two optional features, autocalibration and environment-specific finetuning, using the same dataset. Marker-based motion capture was used as a reference, and the root mean square difference (RMSD) between markerless and marker-based tracking was used as the primary outcome for evaluation of performance. Marker-based tracking is reported to have an accuracy of 2–8°^[29,36]^ and remains the most widely used motion tracking tool in biomechanics. Out of caution and awareness that marker-based analysis is not a perfect ground truth, we used RMSD, rather than RMS error (RMSE), as the primary outcome.

Ground-truth kinematics were obtained using OpenSim’s Inverse Kinematics solver with the Rajagopal musculoskeletal model^[60]^. Kinematic data were processed and standardized across systems as detailed earlier. The RMSD between each markerless method and the marker-based reference served as the primary outcome measure. We employed a linear mixed-effects model^[66]^ to test for differences in RMSD among methods, treating motion-capture method, joint, and activity as fixed effects. Because the RMSD distribution violated residual normality assumptions (Shapiro-Wilk test^[67]^), a log transformation was applied prior to analysis^[16]^. Post-hoc pairwise comparisons were conducted using estimated marginal means, with Bonferroni correction applied to adjust for multiple comparisons.

### Validation in individuals with ACL reconstruction

To evaluate DeepGaitLab’s clinical utility in patients with gait abnormalities, we collected data from 38 individuals at three months following ACLR (25 females and 13 males; age = 22.65 ± 7.36 years; height = 1.71 ± 0.11 m; weight = 70.59 ± 13.53 kg) performing a lateral step-down task, after obtaining approval from the Institutional Review Board of the University of Pittsburgh (STUDY22050192) and informed consents. Performance was compared to 23 individuals without mobility impairments from the I-MOVE-23 dataset described above. Video-based kinematics were extracted using DeepGaitLab (two and ten cameras), OpenCap (two cameras), and Theia3D (ten cameras), utilizing the same camera configurations described above (Fig. 9A), with marker-based kinematics serving as the reference.

We employed a linear mixed-effects model, with limb type (ACLR, healthy) and degree of freedom as fixed effects, and participant as the random effect to account for repeated measurements. Post-hoc pairwise comparisons were conducted using estimated marginal means, with Bonferroni correction for multiple comparisons. This analysis was repeated for both kinematic RMSD and peak hip flexion/extension and adduction/abduction interlimb asymmetries, which are clinically relevant in the ACLR population.

### Validation in individuals with mild cognitive impairment and stroke

After obtaining approval from the Institutional Review Board of the University of Pittsburgh (STUDY19060017) and informed consent, we recruited 19 participants, including 8 individuals with mild cognitive impairment (4 females and 4 males; age = 76.25 ± 4.66 years; height = 1.71 ± 0.12 m; weight = 72.66 ± 13.21 kg) and 11 individuals with stroke (4 females and 7 males; age = 65.55 ± 5.99 years; height = 1.74 ± 0.15 m; weight = 90.82 ± 16.41 kg). Participants with mild cognitive impairment (MCI) completed four split-belt treadmill trials^[3]^. In each trial, participants walked at a fixed and symmetric speed (0.75 m/s) at the beginning and end of the trial, while an asymmetric speed condition was applied during the middle portion (0.5 m/s for the nondominant leg and 1.0 m/s for the dominant leg). Participants with stroke completed six trials, beginning with a baseline trial in which they walked at their self-selected comfortable speed. This trial was used to identify the fast and slow legs. In the remaining trials, participants walked at their comfortable speed at the beginning and end of each trial, with an asymmetric speed condition applied during the middle portion of the trial, using a 2:1 speed ratio. Motion data were recorded with a marker-based motion capture system consisting of 14 IR cameras with 4.0 MP resolution (Vicon, Oxford, UK) at 100 Hz and 8 RGB Sony RX0 II Digital cameras with a resolution of 1920 x 1080 (Sony Group Corporation, Tokyo, Japan) at 120 Hz. The cameras were all arranged around the split-belt treadmill (Fig. 9B).

Step length asymmetry was computed using DeepGaitLab (two-, three-, four-, and eight-camera configurations), OpenCap (two-camera configuration), Theia3D (eight-camera configuration), and marker-based analysis. Heel strike and toe-off events were identified as the positive and negative peaks of the anterior-posterior ankle trajectory relative to the pelvis^[68]^. Step length was then computed for each leg, and step-length asymmetry was defined as the normalized difference between the fast and slow legs^[69]^. To account for individual baseline differences, the mean asymmetry from baseline walking trials was subtracted from all the split-belt trials. Additionally, outlier steps were removed using a z-score thresholding approach^[70]^. Agreement was quantified by computing the RMSD of step-length asymmetry between each markerless method and the marker-based reference as well as intraclass correlation coefficients (ICCs). For method comparisons, the Friedman test was first used to assess overall differences among methods, followed by pairwise Wilcoxon signed-rank tests as post-hoc analyses. To control the family-wise error rate across all the pairwise comparisons, p values were adjusted using the Holm-Bonferroni procedure.

### Inverse dynamics

To evaluate DeepGaitLab’s performance in estimating kinetics, we compared lower-extremity joint moments derived from DeepGaitLab against OpenCap and Theia3D, with marker-based inverse dynamics serving as the reference. As with the kinematic analysis, kinetic data were processed and standardized across systems as detailed above. A subset of twelve healthy participants (6 females and 6 males; age = 38.67 ± 11.15 years; height = 1.71 ± 0.08 m; weight = 80.99 ± 21.03 kg) from the I-MOVE-23 dataset^[16]^, for whom force plate data were collected while performing overground walking, lateral step-down and single-leg squats, were used for the evaluation. Motion data were captured using synchronized marker-based and vision-based systems, as described above. Ground reaction forces were obtained from four floor-embedded force platforms (Bertec, Columbus, OH). All the markerless methods utilized force plate data for inverse dynamics. DeepGaitLab was evaluated at two- and ten-camera configurations, OpenCap was evaluated using two cameras, and Theia3D was evaluated using ten cameras.

Joint moments were computed using OpenSim’s inverse dynamics pipeline with the Rajagopal musculoskeletal model. This approach allows all three markerless systems to be evaluated on the identical musculoskeletal model^[60]^, thereby isolating tracking performance from differences in model definition. Five lower-extremity degrees of freedom were assessed: hip flexion/extension, hip adduction/abduction, hip internal/external rotation, knee flexion/extension, and ankle dorsiflexion/plantarflexion. All the kinematic and kinetic outputs were low-pass filtered using a zero-lag 4th-order Butterworth filter with a 6 Hz cutoff frequency^[71]^. The relative RMSD between each markerless method and the marker-based reference served as the primary outcome measure. We fit a linear mixed-effects model with the markerless method, activity type, and joint degree of freedom as fixed effects and the participant as the random effect. Post-hoc pairwise comparisons were performed using Wilcoxon signed-rank tests with Bonferroni correction.

## Supplementary Material

Supplementary Files

This is a list of supplementary files associated with this preprint. Click to download.

- teaser.mp4
- dataset.mp4

## Figures and Tables

**Figure 7.**
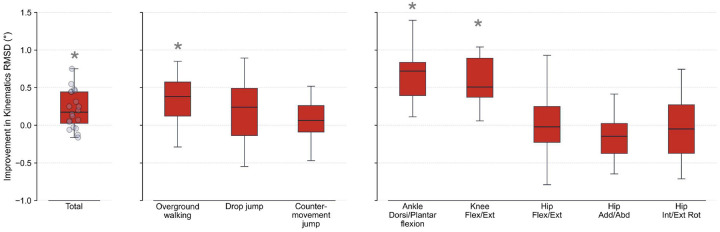
Effect of Environment-Specific Fine-Tuning on Inverse Kinematics Performance. Using the environment-specific synthetic data, the fine-tuned model improved agreement with marker-based motion analysis during overground walking, with the largest gains observed in knee flexion/extension and ankle dorsi/plantarflexion. In each box plot, the horizontal line indicates the median, the box spans the interquartile range (25th to 75th percentiles), and the whiskers represent 1.5 times the interquartile range.

## References

[1] Gage J. R. (1993). Gait analysis. An essential tool in the treatment of cerebral palsy. Clinical Orthopaedics and Related Research, (288), 126–134. 8458125

[2] Winters T. F., Gage J. R., & Hicks R. (1987). Gait patterns in spastic hemiplegia in children and young adults. The Journal of Bone and Joint Surgery. American Volume, 69(3), 437–441. 3818706

[3] Reisman D. S., Wityk R., Silver K., & Bastian A. J. (2007). Locomotor adaptation on a split-belt treadmill can improve walking symmetry post-stroke. Brain, 130(7), 1861–1872. 10.1093/brain/awm035 17405765 PMC2977955

[4] Kotsifaki A., Van Rossom S., Whiteley R., Korakakis V., Bahr R., Sideris V., & Jonkers I. (2022). Single leg vertical jump performance identifies knee function deficits at return to sport after ACL reconstruction in male athletes. British Journal of Sports Medicine, 56(9), 490–498. 10.1136/bjsports-2021-104692 35135826 PMC9016240

[5] Miyazaki T., Wada M., Kawahara H., Sato M., Baba H., & Shimada S. (2002). Dynamic load at baseline can predict radiographic disease progression in medial compartment knee osteoarthritis. Annals of the Rheumatic Diseases, 61(7), 617–622. 10.1136/ard.61.7.617 12079903 PMC1754164

[6] Paterno M. V., Schmitt L. C., Ford K. R., Rauh M. J., Myer G. D., Huang B., & Hewett T. E. (2010). Biomechanical measures during landing and postural stability predict second anterior cruciate ligament injury after anterior cruciate ligament reconstruction and return to sport. The American Journal of Sports Medicine, 38(10), 1968–1978. 10.1177/0363546510376053 20702858 PMC4920967

[7] Verghese J., Lipton R. B., Hall C. B., Kuslansky G., Katz M. J., & Buschke H. (2002). Abnormality of gait as a predictor of non-alzheimer’s dementia. New England Journal of Medicine, 347(22), 1761–1768. 10.1056/NEJMoa020441 12456852

[8] Ruth P. S., Uhlrich S. D., De Monts C., Falisse A., Muccini J., Covitz S., Vogt-Domke S., Day J., Duong T., & Delp S. L. (2025). Video-Based Biomechanical Analysis Captures Disease-Specific Movement Signatures of Different Neuromuscular Diseases. NEJM AI, 2(9). 10.1056/AIoa2401137 PMC12416922 40926960

[9] Kidziński Ł., Yang B., Hicks J. L., Rajagopal A., Delp S. L., & Schwartz M. H. (2020). Deep neural networks enable quantitative movement analysis using single-camera videos. Nature Communications, 11(1), 4054. 10.1038/s41467-020-17807-z PMC7426855 32792511

[10] Stenum J., Hsu M. M., Pantelyat A. Y., & Roemmich R. T. (2024). Clinical gait analysis using video-based pose estimation: Multiple perspectives, clinical populations, and measuring change. PLOS Digital Health, 3(3), e0000467. 10.1371/journal.pdig.0000467 38530801 PMC10965062

[11] Uhlrich S. D., Falisse A., Kidziński Ł., Muccini J., Ko M., Chaudhari A. S., Hicks J. L., & Delp S. L. (2023). OpenCap: Human movement dynamics from smartphone videos. PLOS Computational Biology, 19(10), e1011462. 10.1371/journal.pcbi.1011462 37856442 PMC10586693

[12] Kanko R. M., Laende E. K., Davis E. M., Selbie W. S., & Deluzio K. J. (2021). Concurrent assessment of gait kinematics using marker-based and markerless motion capture. Journal of Biomechanics, 127, 110665. 10.1016/j.jbiomech.2021.110665 34380101

[13] Kanazawa A., Black M. J., Jacobs D. W., & Malik J. (2018). End-to-end recovery of human shape and pose. IEEE Conference on Computer Vision and Pattern Recognition (CVPR), 7122–7131.

[14] Shin S., Kim J., Halilaj E., & Black M. J. (2024). WHAM: Reconstructing world-grounded humans with accurate 3D motion. IEEE/CVF Conference on Computer Vision and Pattern Recognition (CVPR), 2070–2080.

[15] Yang X., Kukreja D., Pinkus D., Sagar A., Fan T., Park J., Shin S., Cao J., Liu J., Ugrinovic N., Feiszli M., Malik J., Dollar P., & Kitani K. (2026). SAM 3D Body: Robust full-body human mesh recovery (Version 1). arXiv. 10.48550/ARXIV.2602.15989

[16] Li Z., Shin S., Phan V., Meinders E., & Halilaj E. (2025). Impact of multi-view fusion and biomechanical modeling on markerless motion tracking. IEEE Transactions on Biomedical Engineering, 1–10. 10.1109/TBME.2025.3622032 PMC13389856 41091616

[17] Ye V., Pavlakos G., Malik J., & Kanazawa A. (2023, June). Decoupling human and camera motion from videos in the wild. IEEE Conference on Computer Vision and Pattern Recognition (CVPR).

[18] Kocabas M., Yuan Y., Molchanov P., Guo Y., Black M., Hilliges O., Kautz J., & Iqbal U. (2024, March). PACE: Human and camera motion estimation from in-the-wild videos. International Conference on 3D Vision (3DV).

[19] Wang Y., Wang Z., Liu L., & Daniilidis K. (2025). TRAM: Global trajectory and motion of 3D humans from in-the-wild videos. In Leonardis A., Ricci E., Roth S., Russakovsky O., Sattler T., & Varol G. (Eds.), European Conference on Computer Vision (ECCV) (Vol. 15069, pp. 467–487). Springer Nature Switzerland. 10.1007/978-3-031-73247-8_27

[20] Black M. J., Patel P., Tesch J., & Yang J. (2023). BEDLAM: A synthetic dataset of bodies exhibiting detailed lifelike animated motion. Proceedings IEEE/CVF Conf. on Computer Vision and Pattern Recognition (CVPR), 8726–8737.

[21] Tesch J., Becherini G., Achar P., Yiannakidis A., Kocabas M., Patel P., & Black M. J. (2025). BEDLAM2.0: Synthetic humans and cameras in motion. The Thirty-Ninth Annual Conference on Neural Information Processing Systems Datasets and Benchmarks Track (NerIPS).

[22] Hewitt C., Saleh F., Aliakbarian S., Petikam L., Rezaeifar S., Florentin L., Hosenie Z., Cashman T. J., Valentin J., Cosker D., & Baltrušaitis T. (2024). Look Ma, no markers: Holistic performance capture without the hassle. ACM Transactions on Graphics (TOG), 43(6).

[23] Iskakov K., Burkov E., Lempitsky V., & Malkov Y. (2019). Learnable triangulation of human pose. International Conference on Computer Vision (ICCV).

[24] Cuevas-Velasquez H., Yiannakidis A., Shin S., Becherini G., Höschle M., Tesch J., Obersat T., Alexiadis T., Halilaj E., & Black M. J. (2025). MAMMA: Markerless & automatic multi-person motion action capture (arXiv:2506.13040). arXiv. 10.48550/arXiv.2506.13040

[25] Delp S. L., Anderson F. C., Arnold A. S., Loan P., Habib A., John C. T., Guendelman E., & Thelen D. G. (2007). OpenSim: Open-source software to create and analyze dynamic simulations of movement. IEEE Transactions on Biomedical Engineering, 54(11), 1940–1950. 10.1109/TBME.2007.901024 18018689

[26] Seth A., Hicks J. L., Uchida T. K., Habib A., Dembia C. L., Dunne J. J., Ong C. F., DeMers M. S., Rajagopal A., Millard M., Hamner S. R., Arnold E. M., Yong J. R., Lakshmikanth S. K., Sherman M. A., Ku J. P., & Delp S. L. (2018). OpenSim: Simulating musculoskeletal dynamics and neuromuscular control to study human and animal movement. PLOS Computational Biology, 14(7), e1006223. 10.1371/journal.pcbi.1006223 30048444 PMC6061994

[27] Grood E. S., & Suntay W. J. (1983). A joint coordinate system for the clinical description of three-dimensional motions: Application to the knee. Journal of Biomechanical Engineering, 105(2), 136–144. 10.1115/1.3138397 6865355

[28] Li Z., Meinders E., Li S., Shin S., L. Sprague A., J. Irrgang J., Volker M., & Halilaj E. (n.d.). Clinical videos enable digital assessment of functional recovery and prediction of long-term outcomes after anterior cruciate ligament reconstruction. Npj Digital Medicine, Under Review.

[29] Miranda D. L., Rainbow M. J., Crisco J. J., & Fleming B. C. (2013). Kinematic differences between optical motion capture and biplanar videoradiography during a jump–cut maneuver. Journal of Biomechanics, 46(3), 567–573. 10.1016/j.jbiomech.2012.09.023 23084785 PMC3551998

[30] Reinschmidt C., Van Den Bogert A. J., Nigg B. M., Lundberg A., & Murphy N. (1997). Effect of skin movement on the analysis of skeletal knee joint motion during running. Journal of Biomechanics, 30(7), 729–732. 10.1016/S0021-9290(97)00001-8 9239553

[31] Shin S., Li Z., & Halilaj E. (2023). Markerless motion tracking with noisy video and IMU data. IEEE Transactions on Biomedical Engineering, 70(11), 3082–3092. 10.1109/TBME.2023.3275775 37171931

[32] Cao Z., Hidalgo G., Simon T., Wei S.-E., & Sheikh Y. (2021). OpenPose: Realtime multi-person 2D pose estimation using part affinity fields. IEEE Transactions on Pattern Analysis and Machine Intelligence, 43(1), 172–186. 10.1109/TPAMI.2019.2929257 31331883

[33] Sun K., Xiao B., Liu D., & Wang J. (2019). Deep high-resolution representation learning for human pose estimation. IEEE Conference on Computer Vision and Pattern Recognition (CVPR).

[34] Lafortune M. A., Cavanagh P. R., Sommer H. J., & Kalenak A. (1992). Three-dimensional kinematics of the human knee during walking. Journal of Biomechanics, 25(4), 347–357. 10.1016/0021-9290(92)90254-X 1583014

[35] Nester C., Jones R. K., Liu A., Howard D., Lundberg A., Arndt A., Lundgren P., Stacoff A., & Wolf P. (2007). Foot kinematics during walking measured using bone and surface mounted markers. Journal of Biomechanics, 40(15), 3412–3423. 10.1016/j.jbiomech.2007.05.019 17631298

[36] Benoit D. L., Ramsey D. K., Lamontagne M., Xu L., Wretenberg P., & Renström P. (2006). Effect of skin movement artifact on knee kinematics during gait and cutting motions measured in vivo. Gait & Posture, 24(2), 152–164. 10.1016/j.gaitpost.2005.04.012 16260140

[37] Phan V., Li Z., Meinders E., Gale T., Anderst W., Ng-Thow-Hing J., Khandan A., & Halilaj E. (n.d.). Inertial motion tracking matches marker-based tracking accuracy: Rethinking modeling approaches toward future progress. Nature Communications, Under Revision.

[38] McFadden C., Daniels K., & Strike S. (2020). The sensitivity of joint kinematics and kinetics to marker placement during a change of direction task. Journal of Biomechanics, 101, 109635. 10.1016/j.jbiomech.2020.109635 32067756

[39] Riemer R., Hsiao-Wecksler E. T., & Zhang X. (2008). Uncertainties in inverse dynamics solutions: A comprehensive analysis and an application to gait. Gait & Posture, 27(4), 578–588. 10.1016/j.gaitpost.2007.07.012 17889542

[40] Holden J. P., Orsini J. A., Siegel K. L., Kepple T. M., Gerber L. H., & Stanhope S. J. (1997). Surface movement errors in shank kinematics and knee kinetics during gait. Gait & Posture, 5(3), 217–227. 10.1016/S0966-6362(96)01088-0

[41] Kadaba M. P., Ramakrishnan H. K., Wootten M. E., Gainey J., Gorton G., & Cochran G. V. B. (1989). Repeatability of kinematic, kinetic, and electromyographic data in normal adult gait. Journal of Orthopaedic Research, 7(6), 849–860. 10.1002/jor.1100070611 2795325

[42] Walter J. P., D’Lima D. D., Colwell C. W., & Fregly B. J. (2010). Decreased knee adduction moment does not guarantee decreased medial contact force during gait. Journal of Orthopaedic Research, 28(10), 1348–1354. 10.1002/jor.21142 20839320 PMC2984615

[43] Stagni R., Leardini A., Cappozzo A., Grazia Benedetti M., & Cappello A. (2000). Effects of hip joint centre mislocation on gait analysis results. Journal of Biomechanics, 33(11), 1479–1487. 10.1016/S0021-9290(00)00093-2 10940407

[44] Loper M., Mahmood N., Romero J., Pons-Moll G., & Black M. J. (2015). SMPL: A skinned multi-person linear model. ACM Transactions on Graphics (Proceedings on SIGGRAPH Asia), 34(6), 248:1–248:16.

[45] Pavlakos G., Choutas V., Ghorbani N., Bolkart T., Osman A. A. A., Tzionas D., & Black M. J. (2019). Expressive body capture: 3D hands, face, and body from a single image. IEEE Conference on Computer Vision and Pattern Recognition (CVPR), 10975–10985.

[46] Varghese R., & M. S. (2024). YOLOv8: A novel object detection algorithm with enhanced performance and robustness. 2024 International Conference on Advances in Data Engineering and Intelligent Computing Systems (ADICS), 1–6. 10.1109/ADICS58448.2024.10533619

[47] Yang C.-Y., Huang H.-W., Chai W., Jiang Z., & Hwang J.-N. (2024). SAMURAI: Adapting segment anything model for zero-shot visual tracking with motion-aware memory. https://arxiv.org/abs/2411.11922

[48] Vaswani A., Shazeer N., Parmar N., Uszkoreit J., Jones L., Gomez A. N., Kaiser Ł. ukasz, & Polosukhin I. (2017). Attention is all you need. In Guyon I., Luxburg U. V., Bengio S., Wallach H., Fergus R., Vishwanathan S., & Garnett R. (Eds.), Advances in Neural Information Processing Systems (Vol. 30). Curran Associates, Inc. https://proceedings.neurips.cc/paper_files/paper/2017/file/3f5ee243547dee91fbd053c1c4a845aa-Paper.pdf

[49] Kolesnikov A., Dosovitskiy A., Weissenborn D., Heigold G., Uszkoreit J., Beyer L., Minderer M., Dehghani M., Houlsby N., Gelly S., Unterthiner T., & Zhai X. (2021). An image is worth 16x16 words: Transformers for image recognition at scale. International Conference on Learning Representation (ICLR).

[50] Lin T.-Y., Maire M., Belongie S., Bourdev L., Girshick R., Hays J., Perona P., Ramanan D., Zitnick C. L., & Dollár P. (2014). Microsoft COCO: Common objects in context. 740–755.

[51] Andriluka M., Pishchulin L., Gehler P., & Schiele B. (2014). 2D Human Pose Estimation: New Benchmark and State of the Art Analysis. IEEE Conference on Computer Vision and Pattern Recognition (CVPR), 3686–3693.

[52] Wu J., Zheng H., Zhao B., Li Y., Yan B., Liang R., Wang W., Zhou S., Lin G., Fu Y., & others. (2019). Large-scale datasets for going deeper in image understanding. 2019 IEEE International Conference on Multimedia and Expo (ICME), 1480–1485.

[53] Mahmood N., Ghorbani N., Troje N. F., Pons-Moll G., & Black M. J. (2019). AMASS: Archive of motion capture as surface shapes. International Conference on Computer Vision (ICCV), 5442–5451.

[54] Loper M. M., Mahmood N., & Black M. J. (2014). MoSh: Motion and shape capture from sparse markers. ACM Transactions on Graphics, (Proc. SIGGRAPH Asia), 33(6), 220:1–220:13. 10.1145/2661229.2661273

[55] Xu Y., Zhang J., Zhang Q., & Tao D. (2022). ViTPose+: Vision transformer foundation model for generic body pose estimation. arXiv Preprint arXiv:2212.04246. 10.1109/TPAMI.2023.3330016 37922160

[56] Xu Y., Zhang J., Zhang Q., & Tao D. (2022). ViTPose: Simple vision transformer baselines for human pose estimation. Advances in Neural Information Processing Systems (NeurIPS).

[57] Hartley R., & Zisserman A. (2004). Multiple view geometry in computer vision (2nd ed.). Cambridge University Press. 10.1017/CBO9780511811685

[58] Schreven S., Beek P. J., & Smeets J. B. J. (2015). Optimising filtering parameters for a 3D motion analysis system. Journal of Electromyography and Kinesiology, 25(5), 808–814. 10.1016/j.jelekin.2015.06.004 26159504

[59] Crenna F., Rossi G. B., & Berardengo M. (2021). Filtering biomechanical signals in movement analysis. Sensors, 21(13), 4580. 10.3390/s21134580 34283131 PMC8271607

[60] Rajagopal A., Dembia C. L., DeMers M. S., Delp D. D., Hicks J. L., & Delp S. L. (2016). Full-body musculoskeletal model for muscle-driven simulation of human gait. IEEE Transactions on Biomedical Engineering, 63(10), 2068–2079. 10.1109/TBME.2016.2586891 27392337 PMC5507211

[61] Leardini A., Sawacha Z., Paolini G., Ingrosso S., Nativo R., & Benedetti M. G. (2007). A new anatomically based protocol for gait analysis in children. Gait & Posture, 26(4), 560–571. 10.1016/j.gaitpost.2006.12.018 17291764

[62] Javerliat C., Raimbaud P., & Lavoué G. (2025). Kineo: Calibration-free metric motion capture from sparse RGB cameras. Arxiv Preprint. https://arxiv.org/abs/2510.24464

[63] Triggs B., McLauchlan P. F., Hartley R. I., & Fitzgibbon A. W. (1999). Bundle adjustment—A modern synthesis. Proceedings of the International Workshop on Vision Algorithms: Theory and Practice, ICCV *’* 99, 298–372.

[64] Schönberger J. L., & Frahm J.-M. (2016). Structure-from-motion revisited. IEEE/CVF Conference on Computer Vision and Pattern Recognition (CVPR).

[65] Coll I., Mavor M. P., Karakolis T., Graham R. B., & Clouthier A. L. (2025). Validation of markerless motion capture for soldier movement patterns assessment under varying body-borne loads. Annals of Biomedical Engineering, 53(2), 358–370. 10.1007/s10439-024-03622-w 39375307

[66] Oberg A. L., & Mahoney D. W. (2007). Linear mixed effects models. In Ambrosius W. T. (Ed.), Topics in Biostatistics (Vol. 404, pp. 213–234). Humana Press. 10.1007/978-1-59745-530-5_11 18450052

[67] Shapiro S. S., & Wilk M. B. (1965). An analysis of variance test for normality (complete samples). Biometrika, 52(3/4), 591. 10.2307/2333709

[68] Stenum J., Rossi C., & Roemmich R. T. (2021). Two-dimensional video-based analysis of human gait using pose estimation. PLOS Computational Biology, 17(4), e1008935. 10.1371/journal.pcbi.1008935 33891585 PMC8099131

[69] Kambic R. E., Roemmich R. T., & Bastian A. J. (2023). Joint-level coordination patterns for split-belt walking across different speed ratios. Journal of Neurophysiology, 129(5), 969–983. 10.1152/jn.00323.2021 36988216 PMC10125032

[70] Zhou L., Fischer E., Brahms C. M., Granacher U., & Arnrich B. (2023). DUO-GAIT: A gait dataset for walking under dual-task and fatigue conditions with inertial measurement units. Scientific Data, 10(1), 543. 10.1038/s41597-023-02391-w 37604913 PMC10442385

[71] Derrick T. R., Van Den Bogert A. J., Cereatti A., Dumas R., Fantozzi S., & Leardini A. (2020). ISB recommendations on the reporting of intersegmental forces and moments during human motion analysis. Journal of Biomechanics, 99, 109533. 10.1016/j.jbiomech.2019.109533 31791632

